# Melatonin Lowers HIF-1α Content in Human Proximal Tubular Cells (HK-2) Due to Preventing Its Deacetylation by Sirtuin 1

**DOI:** 10.3389/fphys.2020.572911

**Published:** 2021-01-14

**Authors:** Aleksandra Owczarek, Katarzyna B. Gieczewska, Marta Polanska, Bohdan Paterczyk, Andrzej Gruza, Katarzyna Winiarska

**Affiliations:** ^1^Department of Metabolic Regulation, Faculty of Biology, Institute of Biochemistry, University of Warsaw, Warsaw, Poland; ^2^Department of Plant Anatomy and Cytology, Faculty of Biology, Institute of Experimental Plant Biology and Biotechnology, University of Warsaw, Warsaw, Poland; ^3^Department of Animal Physiology, Faculty of Biology, Institute of Functional Biology and Ecology, University of Warsaw, Warsaw, Poland; ^4^Laboratory of Electron and Confocal Microscopy, Faculty of Biology, University of Warsaw, Warsaw, Poland

**Keywords:** melatonin, hypoxia-inducible factor-1, sirtuin 1, hypoxia, HK-2 cells, kidney

## Abstract

Although melatonin is widely known for its nephroprotective properties, there are no reports clearly pointing at its impact on the activity of hypoxia-inducible factor-1 (HIF-1), the main mediator of metabolic responses to hypoxia, in kidneys. The aim of the present study was to elucidate how melatonin affects the expression of the regulatory subunit HIF-1α in renal proximal tubules. HK-2 cells, immortalized human proximal tubular cells, were cultured under hypoxic conditions (1% O_2_). Melatonin was applied at 100 μM concentration. Protein and mRNA contents were determined by Western blot and RT-qPCR, respectively. HIF-1α acetylation level was established by means of immunoprecipitation followed by Western blot. Melatonin receptors MT1 and MT2 localization in HK-2 cells was visualized using immunofluorescence confocal analysis. It was found that melatonin in HK-2 cells (1) lowered HIF-1α protein, but not mRNA, content; (2) attenuated expression of HIF-1 target genes; (3) increased HIF-1α acetylation level; and (4) diminished sirtuin 1 expression (both protein and mRNA). Sirtuin 1 involvement in the regulation of HIF-1α level was confirmed applying cells with silenced *Sirt1* gene. Moreover, the presence of membrane MT1 and MT2 receptors was identified in HK-2 cells and their ligand, ramelteon, turned out to mimic melatonin action on both HIF-1α and sirtuin 1 levels. Thus, it is concluded that the mechanism of melatonin-evoked decline in HIF-1α content in renal proximal tubular cells involves increased acetylation of this subunit which results from the attenuated expression of sirtuin 1, an enzyme reported to deacetylate HIF-1α. This observation provides a new insight to the understanding of melatonin action in kidneys.

## Introduction

Hypoxia-inducible factors (HIF’s), HIF-1, HIF-2, and HIF-3, are the main mediators of metabolic responses to the state of hypoxia. Moreover, the abnormal activity of HIF’s seems to be of crucial importance in the pathogenesis of diseases, including cancers and nephropathies (Albadari et al., 2019; Shu et al., 2019).

The most important in renal proximal tubules is HIF-1 (Nangaku et al., 2013; Shu et al., 2019), a heterodimer composed of subunits HIF-1α and HIF-1β. Subunit HIF-1β, responsible for the binding of the transcription factor to HRE (hypoxia response elements) sequence of target genes, is expressed constitutively. In contrast, HIF-1α content is strictly controlled by intracellular oxygen concentration. In normoxia prolyl residues in ODD domain (oxygen-dependent degradation domain) of HIF-1α are hydroxylated by prolyl hydroxylases (PHD’s). Then hydroxylated HIF-1α is recognized by the von Hippel–Lindau (VHL) protein, ubiquitinated, and guided to be degraded in the proteasome (Nangaku et al., 2013; Chan et al., 2016; Shu et al., 2019). Because of this mechanism, the functional complex of HIF-1 practically does not occur under conditions of optimal oxygenation. In addition to regulating the stability of the protein by oxygen concentration, the ultimate activity of HIF-1 is also controlled by a variety of other factors, including those affecting transcription, translation initiation, stability and nuclear translocation of HIF-1α subunit and, finally, functional HIF-1 dimer activity [cf. Koyasu et al. (2018) for review].

It was suggested that changes in HIF-1α acetylation level might play an important role in the regulation of its degradation. However, the issue seems to be complicated as there are reports on both stabilizing and destabilizing effects of acetylation. The regulation of HIF-1α acetylation level is attributed to, among others, sirtuins (a family of seven NAD^+^-dependent proteins with deacetylase activity), of which sirtuin 1 (SIRT1) role seems to be the most important (Lim et al., 2010; Joo et al., 2015; Yoon et al., 2015; Dong et al., 2016; Lin et al., 2018; Ryu et al., 2019; Subbaramaiah et al., 2019).

Melatonin (*N*-acetyl-5-methoxytryptamine), a hormone and an antioxidant, is a derivative of tryptophan occurring in all living organisms. In mammals, it is produced primarily in the pineal gland and performs many functions, including the most famous one—the regulation of circadian and seasonal rhythms (Wood and Loudon, 2014). There are also some intriguing reports on melatonin effects on HIF-1. The first observations were made almost exclusively on tumor cell lines—it was found that melatonin lowers the level of HIF-1α, e.g., in gliomas (Zhang et al., 2013), colon cancer cells (HCT116) (Park et al., 2009), and pancreatic cancer cells (PANC-1) (Dai et al., 2008). However, there are also some more recent reports on melatonin action in non-tumor cells (Kudová et al., 2016; Hsu et al., 2017; Lai et al., 2017; Xu et al., 2018). In some of these cases, it was suggested that the decline in HIF-1 content is achieved due to the increased degradation of HIF-1α subunit, but no detailed mechanism of melatonin action on transcription factor HIF-1 has been clarified yet. Similarly, the data on melatonin effect on sirtuins are also not unequivocal, but the most recent data are rather in favor of the increase in their level (Chen et al., 2019; Savran et al., 2019; Xu et al., 2019).

Surprisingly, although the nephroprotective action of melatonin has been widely reported (Hrenak et al., 2015; Promsan and Lungkaphin, 2020), including our previous studies (Winiarska et al., 2006, 2016), there are no data available regarding melatonin effect on HIF-1 in kidneys. Thus, the aim of the present study was to elucidate how melatonin affects HIF-1α expression in renal proximal tubules. The hypothesis to be verified included the involvement of sirtuin 1 and its direct influence on HIF-1α acetylation level.

## Materials and Methods

### Cell Culture

HK-2 cells (CRL-2190; RRID:CVCL_0302), immortalized human proximal tubular cells, originating from ATCC (Manassas, VA, United States) were used throughout the study. The cells were grown at 37°C under the atmosphere of 5% CO_2_ in Keratinocyte Serum-Free Medium supplemented with bovine pituitary extract (BPE; 0.05 mg/ml) and human recombinant epidermal growth factor (EGF; 5 ng/ml) and subcultured at 80% of confluence.

All the experiments were performed on the cells from the 11th passage. Hypoxic conditions (1% O_2_) were provided due to HK-2 cells’ incubation inside InvivO2 400 hypoxia workstation (Baker Ruskinn, Bridgend, United Kingdom). Melatonin and ramelteon were added at concentrations of 100 μM (Derlacz et al., 2005) and 10 nM (Nishiyama and Hirai, 2014), respectively.

### Preparation of Cell Lysates

Lysates were prepared as described previously (Winiarska et al., 2016). Cells cultured under hypoxic conditions were lysed inside hypoxia workstation (cf. section “Cell Culture”). Protein content was measured spectrophotometrically according to Bradford (1976). Samples were stored at −70°C and just before electrophoresis denatured in Laemmli buffer (5 min, 100°C).

### Immunoprecipitation

Immunoprecipitation was performed with Protein A/G PLUS-Agarose (Santa Cruz Biotechnology), according to the manufacturer’s instructions. Briefly, cell lysates (volumes containing 100 μg of protein; cf. section “Preparation of Cell Lysates”) were incubated with 2 μg of anti-HIF-1α primary antibody (Santa Cruz Biotechnology; cat. no. sc-13515, RRID:AB_627723) for 1 h at 4°C. Then Protein A/G PLUS-Agarose (20 μl) was added and the samples were incubated overnight at 4°C. After centrifugation, the pellets containing protein–antibody–matrix complexes were carefully washed with PBS, denatured in Laemmli buffer (3 min, 100°C), and centrifuged to remove the insoluble matrix. The supernatants dedicated for Western blot analysis were collected and stored at −70°C.

### Western Blot Analysis

Samples (15 μg protein/lane) were applied to polyacrylamide gels [10%; Lonza, Basel, Switzerland, or made according to Rumak et al. (2012)] and electrophoresed, followed by electroblotting to polyvinylidene fluoride membranes (BioRad, Hercules, CA, United States).

After blocking in 5% non-fat dried milk, the membranes were incubated overnight at 4°C with primary antibodies diluted according to the manufacturers’ instructions. Then unbound primary antibodies were removed and the membranes were incubated for 1 h at room temperature with horseradish peroxidase (HRP)–conjugated anti-rabbit IgG secondary antibody (1:1,000; Cell Signaling Technology, cat. no. #7074s, RRID:AB_2099233) or, in case of melatonin receptors MT1 and MT2 detection, with HRP-conjugated anti-mouse IgG secondary antibody (1:10,000; Santa Cruz Biotechnology; cat. no. sc-2005, RRID:AB_631736). HRP-linked mouse anti-β-actin antibody (1:50,000; Abcam; cat. no. ab49900, RRID:AB_867494) was used to verify the equability of samples applied to the gel. The previously stripped membranes were incubated with them for 1 h at room temperature.

Protein detection was performed by the enhanced chemiluminescence (ECL) method using ChemiDoc Imaging System (BioRad, Hercules, CA, United States). The density of the bands was analyzed by Image Lab software (BioRad; RRID:SCR_014210). Electrophoresis and electroblotting were performed using MiniProtean Tetra System and TransBlot System (BioRad), respectively.

### PCR Analysis

Total RNA was extracted with Universal RNA Purification Kit (EURx), according to the manufacturer’s instructions for cell cultures (with DNase digestion), and eluted with 70 μl of water. Directly after the isolation, RNA quality was checked with NanoDrop spectrophotometer (Thermo Scientific, Waltham, MA, United States). RNA was stored at −80°C and thawed only shortly before the experiment. The primers were designed, respectively, according to β-actin—Yu et al. (2014), RPL13A—Palombella et al. (2017), HIF-1α—Coimbra et al. (2004), SIRT1—Ishikawa et al. (2013), pyruvate dehydrogenase kinase 1 (PDK-1)—Zhou et al. (2019), and VEGF—Chen et al. (2016).

Expression analysis was conducted by quantitative PCR with MyGo Pro Real-Time PCR thermocycler (IT-IS International, Middlesbrough, United Kingdom), using SensiFAST SYBR Green MasterMix (Bioline) and recommended thermal profile (45 cycles). After amplification, a melt curve was performed in the 60–95°C range, with 0.5°C steps.

Relative gene expression in each sample was calculated with MyGo Pro analysis software v.3.3. (IT-IS International), normalized to reference genes (β-actin and RPL13A), and scaled to the calibrator sample (obtained in the absence of melatonin). Intra-assay variation was evaluated by calculating SEM errors of sample replicates.

### Gene Silencing

For transient *Sirt1* gene silencing, HK-2 cells were transfected with TransIT-TKO Transfection Reagent (Mirus Bio LLC), according to the manufacturer’s instructions. SignalSilence SirT1 siRNA I (Cell Signaling Technology, cat. no. 12241) was used as target siRNA and applied at 100 nM concentration, as recommended by the manufacturer. SignalSilence Control siRNA (unconjugated) (Cell Signaling Technology, cat. no. 6568) served as a negative control. The efficiency of the silencing was confirmed by Western blot analysis (cf. section “Western Blot Analysis”) of SIRT1 protein content in cells lysed 48 h after transfection. There was no difference in SIRT1 expression in intact cells and in cells transfected with negative control siRNA.

### Confocal Immunofluorescence Analysis of MT1 and MT2 Receptor Localization

The localization of MT1 and MT2 receptors in HK-2 cells was determined using indirect immunofluorescence method. Before staining, cells were cultured, as described in section “Cell Culture”, on chambered culture slides (Profilab, Warsaw, Poland), washed briefly in phosphate-buffered saline (PBS), and fixed in 1% paraformaldehyde in PBS for 10 min at room temperature. After fixation, cells were washed in three changes of PBS, permeabilized by incubation of slides in 0.01% Triton X-100 in PBS for 3 min, blocked in 10% normal goat serum (NGS) in PBS for 10 min, and again washed three times in PBS at room temperature. Primary monoclonal antibody MEL-1A/B-R (Santa Cruz Biotechnology, cat. no. sc-398788, RRID:AB_2833090) was applied for detection of MT1 and MT2 receptors. The slides were incubated with the antibody diluted (1:250) in PBS containing 1.5% NGS overnight at 4°C. Next, the specimens were washed in several changes of PBS and incubated for 1 h at room temperature with secondary anti-mouse IgG antibody conjugated with Alexa Fluor 488 (Thermo Fisher Scientific, cat. no. A-11029, RRID:AB_138404), and diluted (1:1,000) in PBS containing 1.5% NGS. After incubation, unbound antibodies were removed by rinsing slides three times in PBS. Finally, the specimens were incubated in nuclear dye (0.05% bisbenzimide in PBS, Hoechst 33342) as histochemical counterstain and washed in several changes of PBS. The stained slides were mounted in Fluoromount-G mounting medium (Southern Biotech). Section analysis and microphotography were performed using a confocal laser microscope Nikon A1R MP (Nikon Corporation, Tokyo, Japan).

### Antibodies and Chemicals

The antibodies originated from the following: Cell Signaling Technology (Danvers, MA, United States)—anti-HIF-1α (cat. no. 36169, RRID:AB_279909, used for Western blot), anti-SIRT1 (cat. no. 2310, RRID:AB_561272), anti-acetylated lysine (cat. no. 9441, RRID:AB_331805), and anti-rabbit IgG (cat. no. 7074s, RRID:AB_2099233); Santa Cruz Biotechnology (Dallas, TX, United States)—anti-HIF-1α (cat. no. sc-13515, RRID:AB_627723, used for immunoprecipitation), MEL-1A/B-R (cat. no. sc-398788, RRID:AB_2833090), and anti-mouse IgG (cat. no. sc-2005, RRID:AB_631736); Abcam (Cambridge, United Kingdom)—anti-beta-actin conjugated with HRP (cat. no. ab49900, RRID:AB_867494); Thermo Fisher Scientific (Waltham, MA, United States)—anti-mouse IgG antibody conjugated with Alexa Fluor 488 (cat. no. A-11029, RRID:AB_138404).

Oligonucleotides: SignalSilence SirT1 siRNA I (cat. no. 12241) and SignalSilence Control siRNA (unconjugated) (cat. no. 6568) were from Cell Signaling Technology.

RNA purification kit and PCR chemicals originated from, respectively, EURx (Gdańsk, Poland) and Bioline (London, United Kingdom). ECL reagent—Westar Supernova was from Cyanagen (Bologna, Italy). A/G PLUS-Agarose was from Santa Cruz Biotechnology. TransIT-TKO Transfection Reagent was from Mirus Bio LLC (Madison, WI, United States). Fluoromount-G mounting medium originated from Southern Biotech (Birmingham, AL, United States). Keratinocyte-SFM (1×) kit was from Gibco (Carlsbad, CA, United States). Melatonin, ramelteon, and all other chemicals were purchased from Sigma Chemicals (St. Louis, MO, United States).

### Expression of Results

The significance of the differences was estimated using ANOVA. Values are expressed as means ± SEM for three to five separate experiments.

## Results

### The Content of HIF-1α Protein but Not of Its mRNA Is Lowered by Melatonin

**FIGURE 1:**
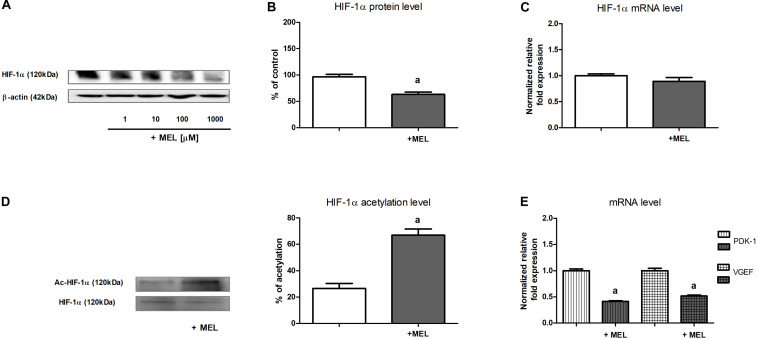
Melatonin effect on HIF-1α expression [panels **(A,B)**—protein, Western blot analysis; panel **(C)**—mRNA, RT-qPCR analysis], HIF-1α acetylation level [panel **(D)**—Western blot analysis preceded with immunoprecipitation], and expression of chosen HIF-1α target genes [panel **(E)**—mRNA, RT-qPCR analysis] in HK-2 cells. Cells were cultured for 24 h under hypoxic (1% O_2_) conditions. Melatonin was applied at 100 μM concentration or at concentrations indicated in the figure. Before Western blot analysis, lysates intended for the determination of HIF-1α acetylation level were immunoprecipitated against HIF-1α. After acetyl-HIF-1α analysis with anti-acetylated-lysine antibodies, the membranes were stripped and then reprobed against HIF-1α. Values are means ± SEM for 3–5 experiments. Statistical significance: ^*a*^*p* < 0.05 vs. corresponding values in the absence of melatonin.

As presented in Figure 1A, the addition of melatonin (at the range of 1–1,000 μM) to experimental media resulted in a concentration-dependent decline in HIF-1α protein content in HK-2 cells cultured under hypoxic (1% O_2_) conditions. Also, 100 μM melatonin, which was applied in the subsequent experiments, lowered HIF-1α protein level by *ca.* 40% (Figure 1B). However, the level of HIF-1α mRNA remained unchanged in the presence of melatonin (Figure 1C), suggesting that melatonin does not directly affect *HIF1A* transcription.

Under normoxic conditions, HIF-1α level was negligible and melatonin did not affect it (data not shown).

### The Level of HIF-1α Acetylation Is Increased in the Presence of Melatonin

In view of the earlier observations (cf. section “The Content of HIF-1α Protein but Not of Its mRNA Is Lowered by Melatonin”), we presumed that melatonin inhibitory action on HIF-1α expression in HK-2 cells involves its impact on the stability of this protein. Because acetylation/deacetylation is considered to be a posttranslational modification that might be of crucial importance for the regulation HIF-1α stability (Lim et al., 2010; Joo et al., 2015; Lin et al., 2018; Ryu et al., 2019), we decided to measure the level of HIF-1α acetylation in the absence and in the presence of melatonin. As shown in Figure 1D, the level of HIF-1α acetylation in cells incubated with 100 μM melatonin was more than two times higher than that in control cells.

### The Expression of HIF-1α Target Genes Is Attenuated in the Presence of Melatonin

We were interested if the melatonin-evoked decline in HIF-1α regulatory subunit content (cf. section “The Content of HIF-1α Protein but Not of Its mRNA Is Lowered by Melatonin”) effectively affected HIF-1 activity in HK-2 cells. Thus, we decided to examine the expression of two genes commonly known to be controlled by HIF-1—encoding pyruvate dehydrogenase kinase 1 (PDK-1) (Zhou et al., 2019) and encoding VEGF (Chen et al., 2016). As presented in Figure 1E, upon the addition of melatonin to the experimental media, mRNA levels of either PDK-1 or VEGF were diminished about two times, clearly suggesting that HIF-1 activity was attenuated.

It is also worth mentioning that hypoxia resulted in a 200% increase in both PDK-1 and VEGF mRNA levels in HK-2 cells, comparing with normoxic conditions (data not shown).

### The Expression of Sirtuin 1 in Hypoxia Is Decreased by Melatonin

**FIGURE 2:**
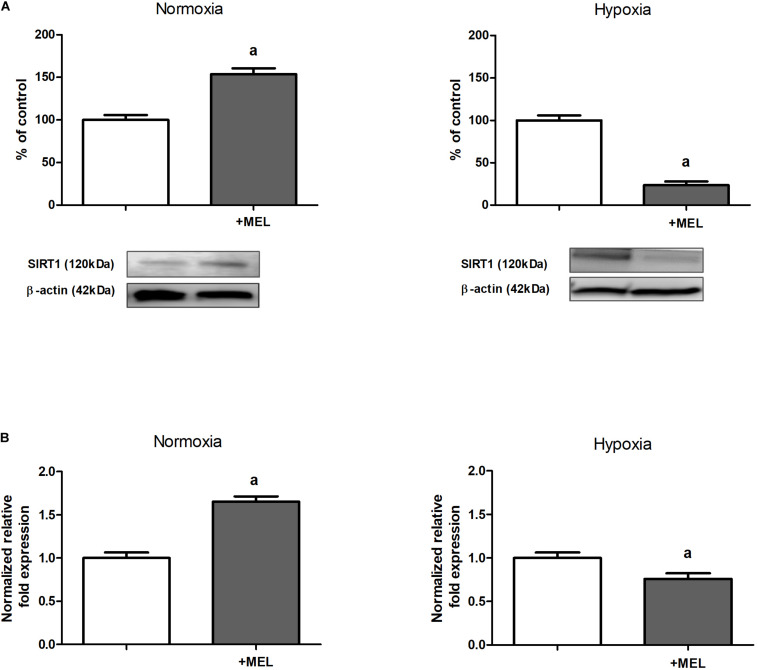
Melatonin effect on sirtuin 1 (SIRT1) expression [panel **(A)**—protein, Western blot analysis; panel **(B)**—mRNA, RT-qPCR analysis] in HK-2 cells. Cells were cultured for 24 h under normoxic or under hypoxic (1% O_2_) conditions in the absence or in the presence of 100 μM melatonin. Values are means ± SEM for 3–5 experiments. Statistical significance: ^*a*^*p* < 0.05 vs. corresponding values in the absence of melatonin.

The next step of our study was finding the mechanism responsible for melatonin-evoked increase in HIF-1α acetylation (cf. section “The Level of HIF-1α Acetylation Is Increased in the Presence of Melatonin”). We turned our attention to sirtuins, particularly to sirtuin 1, known to be able to deacetylate many proteins, including HIF-1α (Joo et al., 2015; Ryu et al., 2019), and we proved that in HK-2 cells cultured under hypoxic conditions, both protein (Figure 2A) and mRNA (Figure 2B) levels of SIRT1 were significantly diminished upon the addition of melatonin to experimental media.

In normoxia, however, supplementation with melatonin resulted in a notable increase in SIRT1 level in HK-2 cells (Figure 2).

### Sirt1 Gene Knockdown Results in Diminished HIF-1α Content

**FIGURE 3:**
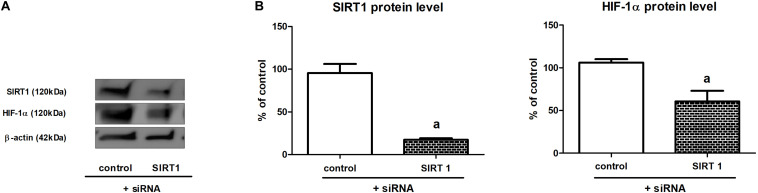
The effect of *Sirt1* gene knockdown on HIF-1α expression in HK-2 cells [panel **(A)**—a sample result of Western blot; panel **(B)**—quantitative analysis]. Cells were cultured for 24 h under hypoxic (1% O_2_) conditions. Gene silencing procedure was performed as described in section “Gene Silencing”. Values are means ± SEM for 3–5 experiments. Statistical significance: ^*a*^*p* < 0.05 vs. corresponding values for cells with no *Sirt1* knockdown.

To confirm that sirtuin 1 is involved in the regulation of HIF-1α content in HK-2 cells incubated under hypoxic conditions, we performed experiments including *Sirt1* gene knockdown, which was achieved due to application of specific siRNA (cf. section “Gene Silencing”). To our satisfaction, we found that cells with silenced *Sirt1* gene (80% knockdown efficiency, as estimated by Western blot analysis) exhibited diminished (by *ca.* 40%) HIF-1α protein level (Figure 3).

### Melatonin Receptors MT1 and MT2 Are Expressed in Renal Proximal Tubules and Mediate Melatonin Inhibitory Effect on HIF-1α Content

**FIGURE 4:**
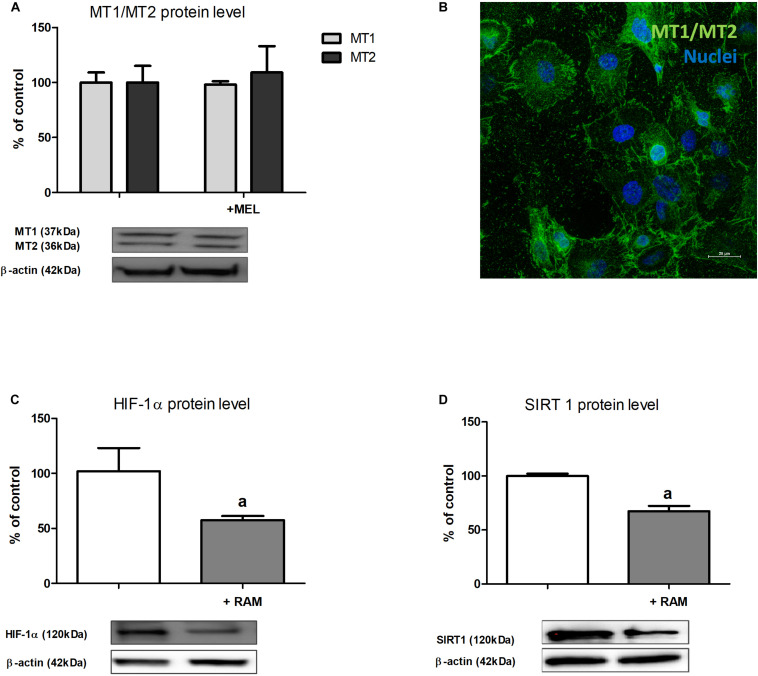
The expression of melatonin membrane receptors MT1 and MT2 in HK-2 cells [panel **(A)**—Western blot analysis; panel **(B)**—confocal immunofluorescence analysis] and the effect of ramelteon (MT1/MT2 receptors ligand) on HIF-1α [panel **(C)**—protein, Western blot analysis] and sirtuin 1 [panel **(D)**—protein, Western blot analysis] expression. Cells were cultured for 24 h under hypoxic (1% O_2_) conditions. Melatonin and ramelteon were applied at 100 μM and 10 nM concentrations, respectively. Cells intended for immunofluorescence analysis were stained as described in section “Confocal Immunofluorescence Analysis of MT1 and MT2 Receptor Localization”. Values are means ± SEM for 3–5 experiments. ^*a*^*p* < 0.05 vs. corresponding values in the absence of ramelteon.

Finally, we decided to check if in renal proximal tubules melatonin might act *via* its membrane receptors and we determined MT1 and MT2 expression in HK-2 cells. Western blot analysis (Figure 4A) proved that MT1 and MT2 are present in HK-2 cells and confocal immunofluorescence analysis (Figure 4B) confirmed their membrane localization. The addition of melatonin to experimental media did not affect MT1 and MT2 expression in HK-2 cells incubated under hypoxic conditions.

To confirm the hypothesis that MT1 and MT2 receptors mediate melatonin inhibitory effect on HIF-1α content, we performed experiments applying ramelteon, a selective agonist of these receptors (Nishiyama and Hirai, 2014). As presented in Figure 4C, the addition of ramelteon to experimental media led to *ca.* 40%, i.e., very similar to that observed in the presence of melatonin (cf. section “The Content of HIF-1α Protein but Not of Its mRNA Is Lowered by Melatonin”), decline in HIF-1α protein content in HK-2 cells incubated in hypoxia. Moreover, upon the addition of ramelteon, a significant decrease in sirtuin 1 protein level was also observed (Figure 4D).

## Discussion

Our present study is the first that reports melatonin inhibitory action on HIF-1 in renal proximal tubules, fixing together the three pieces of the same jigsaw puzzle: (1) melatonin decreases HIF-1α expression; (2) melatonin affects SIRT1 expression; (3) deacetylation catalyzed by SIRT1 may regulate HIF-1α stability. We have demonstrated that in HK-2 cells incubated under hypoxic conditions, melatonin decreases HIF-1α content due to preventing its deacetylation by sirtuin 1.

Melatonin inhibitory effect on HIF-1 has been described mainly in the context of its anticancer action (Dai et al., 2008; Zhang et al., 2013; Paroni et al., 2014; Colombo et al., 2016; Prieto-Domínguez et al., 2017; Wang et al., 2017; Cheng et al., 2019), indicating melatonin’s potent role as a negative regulator of tumor-related angiogenesis. Equally interesting are reports on melatonin’s action on HIF-1 in non-tumor cells. Kudová et al. (2016) found that melatonin promotes cardiomyogenesis of embryonic stem cells *via* lowering HIF-1α content. Hsu et al. (2017) postulated that the mechanism of melatonin’s protective action in liver damaged by trauma-hemorrhage includes normalization of HIF-1α expression. Lai et al. (2017) reported melatonin-evoked decrease in HIF-1α level in retinal epithelial cells and Xu et al. (2018) explained that melatonin prevented pathologic neovascularization and other abnormalities characteristic of oxygen-induced retinopathy *via* inhibition of HIF-1/VEGF pathway. Similarly, Cheng et al. (2019) reported melatonin-induced inhibition of HIF-1 leading to lowered VEGF secretion in HUVECs. Thus, our findings on melatonin action on HIF-1 in renal proximal tubules (cf. Figure 1) are in agreement with the earlier observations. Moreover, it is worth emphasizing that our data clearly indicate that melatonin-evoked decline in regulatory subunit α content results directly in lowered HIF-1 activity, as concluded from the attenuated expression of its target genes, including those encoding VEGF and PDK-1.

Numerous reports indicate melatonin’s stimulatory effect on SIRT1 expression in different types of non-tumor cells (Shah et al., 2017; Chen et al., 2019; Savran et al., 2019; Xu et al., 2019) and tissues, including heart (Favero et al., 2020) and kidneys (Bai et al., 2016; Shi et al., 2019). However, all these studies were conducted in normoxia and it seems likely that melatonin action on sirtuin 1 level may depend on oxygenation conditions. This hypothesis is confirmed by our own observation that HK-2 cells cultured under normoxic conditions also exhibit melatonin-increased sirtuin 1 expression (cf. Figure 2). Interestingly, melatonin-evoked decline in SIRT1 level was found in case of tumor cells (Jung-Hynes et al., 2011; Cheng et al., 2013; Proietti et al., 2014) that also represent abnormal energy metabolism, similar to that characteristic of hypoxic conditions.

The third piece of the jigsaw puzzle is the most interesting but also appears the most disputable. Although there are no doubts that sirtuin 1 is able to deacetylate HIF-1α, as the direct interaction between these two proteins was confirmed by co-immunoprecipitation experiments (Joo et al., 2015; Ryu et al., 2019), the authors argue which is the final effect of lowered HIF-1α acetylation: stabilizing or—just on the contrary—guiding to degradation in proteasome. Our data (cf. Figure 1) suggest that increased acetylation of HIF-α lowers its stability. Moreover, the results of the experiments applying cells with SIRT1 knockdown (cf. Figure 3) confirm the involvement of sirtuin 1. Similar mechanisms were postulated by Joo et al. (2015) and Lin et al. (2018). The first team found that SIRT1 deacetylates HIF-1α and promotes its hypoxic accumulation in HeLa and some other cell lines; the second one found that experimentally decreased activity of sirtuin 1 results in elevated acetylation level and downregulated activity of HIF-1 in retinal vascular endothelial cells. The opposite point of view was presented by Ryu et al. (2019), who postulated that SIRT1-induced deacetylation of HIF-1α has protective effects in aged kidney, and by Lim et al. (2010), who additionally suggested the important role of NAD^+^/NADH ratio in the regulation of HIF-1 activity by sirtuin 1. Thus, it is not easy to interpret such discrepant reports. We could only hypothesize that the final effect of the changes in HIF-1α acetylation on the stability of this protein might depend on additional factors, e.g., oxygenation conditions.

Melatonin’s biological action can be carried out with the involvement of specific receptors. The best characterized, membrane-bound ML1 receptors include two subclasses: MT1 and MT2 (Jockers et al., 2016). ML1 receptors inhibit adenylate cyclase *via* interaction with G proteins, thereby lowering the concentration of cAMP in the cell. Moreover, ML1 receptors can stimulate phospholipase C activity by increasing the intracellular calcium concentration and leading to the protein kinase C activation. The receptors of MT2 subclass can also affect the concentration of cGMP in the cell (Slominski et al., 2012). In kidneys, the presence of both MT1 and MT2 receptors was revealed (Hrenak et al., 2015). Moreover, downregulated expression of MT1 was found to be characteristic of membranous nephropathy, a type of glomerulonephritis (Huang et al., 2018). We confirmed not only the presence of MT1 and MT2 receptors in renal proximal tubules but also their involvement in melatonin inhibitory action on HIF-1 (cf. Figure 4). The results of the experiments with the use of ramelteon (Nishiyama and Hirai, 2014) indicate that MT1/MT2 receptors ligand perfectly mimics melatonin’s effects on both HIF-1α and SIRT1 levels. Interestingly, in agreement with our findings, Sánchez et al. (2018) in their study on hepatocellular carcinoma also reported that MT1/MT2 receptors could mediate in melatonin’s action on sirtuin 1 level.

Melatonin’s nephroprotective properties have been widely discussed, especially in the context of diabetic nephropathy [cf. Promsan and Lungkaphin (2020) and Hrenak et al. (2015) for the most recent reviews], including our previous studies on alloxan diabetic rabbits (model of type 1 diabetes) (Winiarska et al., 2006) and ZDF rats (model of type 2 diabetes) (Winiarska et al., 2016). Although numerous other mechanisms of melatonin action were proposed, its nephroprotective effects have never been attributed to the attenuation of HIF-1 activity. It seems rather surprising as HIF-1 is considered to be one of the most important factors promoting renal fibrosis which is the most common symptom of chronic kidney disease (CKD) (Wei et al., 2020).

Finally, it is worth to be emphasized that in kidneys, the precise regulation of HIF-1 activity is of special importance because they are organs of notably high oxygen demand and, in consequence, extremely susceptible to hypoxia and hypoxic damage which is reported to be responsible for the development of numerous renal pathologies [cf. Liu et al. (2017), Honda et al. (2019), Shu et al. (2019) for review], both in acute kidney injury and in CKD, including diabetic kidney disease. In addition, hypoxia/HIF’s are of special interest due to their role as central mediators of renal tumor risk (Chappell et al., 2019). Thus, factors modulating the expression of regulatory subunit HIF-1α might be interesting as potential therapeutics. Melatonin also seems worth studying in this context.

## Data Availability Statement

The raw data supporting the conclusions of this article will be made available by the authors, without undue reservation.

## Author Contributions

AO and KW together developed the conception of the work. AO and KG performed the experiments and analyzed the data. MP customized the method and performed immunofluorescence staining. BP analyzed the microscopic data. AG performed the experiments. AO prepared the draft article and all of the figures. KW wrote the final version of the manuscript. All authors contributed to the article and approved the submitted version.

## Conflict of Interest

The authors declare that the research was conducted in the absence of any commercial or financial relationships that could be construed as a potential conflict of interest.
